# Safety and Tolerability of Burst-Cycling Deep Brain Stimulation for Freezing of Gait in Parkinson’s Disease

**DOI:** 10.3389/fnhum.2021.651168

**Published:** 2021-04-26

**Authors:** Joshua K. Wong, Wei Hu, Ryan Barmore, Janine Lopes, Kathryn Moore, Joseph Legacy, Parisa Tahafchi, Zachary Jackson, Jack W. Judy, Robert S. Raike, Anson Wang, Takashi Tsuboi, Michael S. Okun, Leonardo Almeida

**Affiliations:** ^1^Department of Neurology, Norman Fixel Institute for Neurological Diseases, University of Florida, Gainesville, FL, United States; ^2^Banner Health Physicians Colorado, Loveland, CO, United States; ^3^Department of Electrical and Computer Engineering, University of Florida, Gainesville, FL, United States; ^4^Nanoscience Institute for Medical and Engineering Technology, University of Florida, Gainesville, FL, United States; ^5^Restorative Therapies Group Implantables, Research and Core Technology, Medtronic, Minneapolis, MN, United States; ^6^Department of Neurology, Nagoya University Graduate School of Medicine, Nagoya, Japan

**Keywords:** deep brain stimulation, freezing of gait, Parkinson’s disease, burst cycling, GPi, STN, globus pallidum interna, subthalamic nucleus

## Abstract

**Background**: Freezing of gait (FOG) is a common symptom in Parkinson’s disease (PD) and can be difficult to treat with dopaminergic medications or with deep brain stimulation (DBS). Novel stimulation paradigms have been proposed to address suboptimal responses to conventional DBS programming methods. Burst-cycling deep brain stimulation (BCDBS) delivers current in various frequencies of bursts (e.g., 4, 10, or 15 Hz), while maintaining an intra-burst frequency identical to conventional DBS.

**Objective**: To evaluate the safety and tolerability of BCDBS in PD patients with FOG.

**Methods**: Ten PD subjects with STN or GPi DBS and complaints of FOG were recruited for this single center, single blinded within-subject crossover study. For each subject, we compared 4, 10, and 15 Hz BCDBS to conventional DBS during the PD medication-OFF state.

**Results**: There were no serious adverse events with BCDBS. It was feasible and straightforward to program BCDBS in the clinic setting. The benefit was comparable to conventional DBS in measures of FOG, functional mobility and in PD motor symptoms. BCDBS had lower battery consumption when compared to conventional DBS.

**Conclusions**: BCDBS was feasible, safe and well tolerated and it has potential to be a viable future DBS programming strategy.

## Introduction

Freezing of gait (FOG) is a common symptom in Parkinson’s disease (PD) and tends to increase in prevalence with disease duration (Giladi et al., 2001). Although the underlying mechanism is not well understood, FOG has a complex pathophysiology that can be provoked by a variety of internal and external stimuli (Gilat et al., 2018). Historically, FOG can be dopamine responsive or “dopamine-resistant” and has been challenging to address (Okuma, 2014). FOG therefore can be a disabling manifestation of PD and can severely impact quality of life. Although deep brain stimulation (DBS) has emerged as a reliable treatment option for motor fluctuations, dyskinesia, and tremor, there has been mixed success when applied to FOG (Weaver, 2009). The subthalamic nucleus (STN), globus pallidus internus (GPi), and pedunculopontine nucleus (PPN) have all been trialed for FOG (Nilsson et al., 2009; Rocchi et al., 2012; Schrader et al., 2013; Vercruysse et al., 2014; Welter et al., 2015; Kim et al., 2019). Exploratory studies have observed that axial symptoms such as FOG are less responsive to conventional high-frequency DBS (>100 Hz; Gervais-Bernard et al., 2009; Fasano et al., 2010, 2015). However, several small studies have found that low-frequency stimulation (<100 Hz) can improve gait in PD (Moreau et al., 2008; Xie et al., 2016). Additionally, physiology studies have shown a dynamic temporal relationship between intrinsic basal ganglia oscillations and the various components of gait (Fischer et al., 2018, 2020). Novel stimulation paradigms have been proposed as a possible alternatives to conventional DBS that may improve the suboptimal responses to classic DBS approaches (Akbar et al., 2016). Several previous studies by our group have demonstrated that firmware updates of DBS pulse shapes and patterns can be safe and well-tolerated (Almeida et al., 2017; De Jesus et al., 2018). We conducted a safety and tolerability trial of a temporally focused pattern of stimulation called burst-cycling DBS (BCDBS) applied to PD subjects with chronically implanted unilateral or bilateral STN or GPi DBS.

## Materials and Methods

The study protocol was approved by the Institutional Review Board (IRB201602593) at the University of Florida (UF). Ten PD subjects with complaints of FOG interviewed by a movement-disorders neurologist in clinic setting were recruited for this study. The primary outcome measure of the study was the safety of BCDBS as determined *via* first hand observation by the examiner. Poor tolerability was defined as the occurrence of any stimulation induced side effects that required cessation of BCDBS programming. Secondary outcome measures included assessment of BCDBS on FOG, gait metrics, and motor symptom severity *via* the Unified PD Rating Scale (UPDRS).

Inclusion criteria for this study were: (1) a diagnosis of PD as defined by UK Brain Bank Criteria; (2) complaints of FOG at home; (3) chronic and optimized DBS; and (4) Medtronic DBS lead (model 3387) and implantable pulse generator (IPG) that is either Activa SC, PC, or RC (Medtronic, Minneapolis, MN, USA; Gelb et al., 1999). “Chronic and optimized” DBS in this study is defined as having the same DBS settings for a duration of at least 6 months. Exclusion criteria in this study were: (1) any other previous neurological surgery; (2) DBS hardware other than the Medtronic system; (3) baseline utilization of complex DBS programming settings such as interleaving stimulation; and (4) suspicion of other neurologic diagnoses such as Parkinsonism, Atypical parkinsonism, or Alzheimer’s disease.

**Figure 1:**
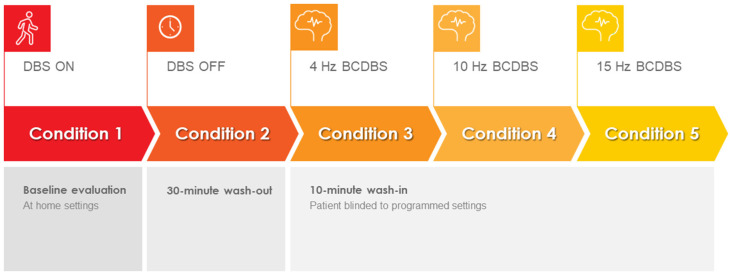
Burst cycling deep brain stimulation (BCDBS) testing protocol: subjects were tested under five sequential conditions: (1) baseline, (2) 30 min after turning the DBS OFF, (3) 10 min after initiating 4-Hz BCDBS, (4) 10 min after initiating 10-Hz BCDBS, and (5) 10 min after initiating 15-Hz BCDBS.

**Figure 2:**
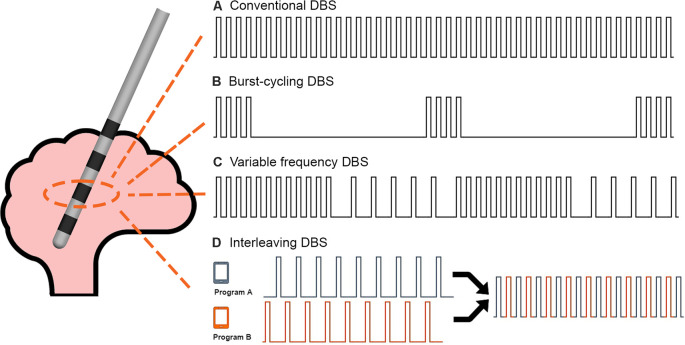
DBS stimulation paradigms: a comparison of the stimulation delivery patterns between **(A)** Conventional DBS, **(B)** Burst-cycling DBS, **(C)** Variable frequency DBS, and **(D)** Interleaving DBS is shown. Burst-cycling DBS delivers four pulses at the same intra-burst frequency as conventional DBS but with an inter-burst frequency of 4, 10, or 15 Hz. The illustrated example shows 4-Hz burst-cycling DBS.

The study was conducted during a 1-day office visit. The study visit lasted approximately 6 h and included clinical testing under five different DBS programming conditions. Specifically, the subjects in this study presented to the clinic in the medication-OFF state after a 12-h overnight withdrawal of dopaminergic medications. Upon arrival, the patient IPG was interrogated using the standard Medtronic clinician programmer. The interrogation was used to verify hardware integrity. The IPG was then flashed to a Medtronic research firmware using the Medtronic Neuro Research Programmer (NRP) tool (Akbar et al., 2016). The NRP tool was then used to program the IPG to the baseline home settings for each subject (i.e., active contacts, voltage, pulse width, and frequency). Each subject was then tested in a single blinded fashion under five different programming conditions: (1) baseline home settings; (2) 30 min after turning the DBS off (i.e., a 30-min “wash-out” period; (3) 4-Hz burst-cycling stimulation; (4) 10-Hz burst-cycling stimulation; and (5) 15-Hz burst-cycling stimulation. There was a 10-min wash-in period between the different burst-cycle settings where the patients were instructed to rest while receiving BCDBS. During BCDBS, the voltage and pulse width were kept at baseline settings. The testing protocol can be seen in Figure 1. A visual explanation of BCDBS and comparison to other stimulation paradigms is shown in Figure 2. The BCDBS paradigm can be applied to the common programming frequencies conventionally available in the Medtronic clinician programmer.

For each testing condition, the subject was assessed for the following: e modified video UPDRS part III, 3-m timed up and go, freezing during clinic walking path, and a Zeno Walkway gait analysis (i.e., ProtoKinetics LLC, Havertown, PA). The clinic walking path was approximately a 100-foot path that included a wide hallway, a narrow hallway, one right turn and one left turn. During the Zeno Walkway gait analysis, patients were instructed to walk down a 26-foot-long by 2-foot-wide pressure sensing mat at their usual preferred pace, turn around and walk back to the starting position. Stimulation induced side effects were assessed for at the beginning and end of each testing condition interval. A checklist of common stimulation induced side effects as well as open-ended questioning was used to assess for side effects during BCDBS. Motor and gait assessments were videotaped and independently evaluated by two blinded movement-disorders neurologists. During the clinic walk, the movement-disorders neurologists were instructed to identify and count the number of definite freezing episodes observed during the video. For each of the three burst-cycling stimulation programs, clinical assessments were conducted after allowing for a 10-min stimulation wash-in period. At the end of the study, the NRP tool was used to unload the research firmware and restore the factory default settings. The standard Medtronic clinician programmer was then used to restore baseline home settings for each subject.

Given the low sample size and the fact that this was a safety and tolerability study, we assumed a non-parametric distribution of data. Each of the clinical metrics were organized by the five testing conditions and were imported into SPSS (version 25; SPSS, Inc., Chicago, IL, USA) for analysis. The five conditions were tested for differences using a non-parametric analysis of variance (Kruskal–Wallis test) using a *p* = 0.05 as the threshold for statistical significance. Wilcoxon’s signed rank test was similarly used for non-parametric comparisons of dependent measurements.

## Results

Ten PD subjects (eight men, two women) were recruited for the study. The median (IQR) age was 66 (64–75) years. The median (IQR) disease duration was 11 (9–16) years and time since DBS surgery was 19 (11–51) months. The median (IQR) UPDRS part II Freezing when Walking (Item 14) and Walking (Item 15) scores were 2 (2–3) and 2 (0.75–2.3), respectively. The median (IQR) Hoehn and Yahr scale and Dementia Rating Scale (2nd Edition) were 2.8 (2.4–3) and 135 (133–139), respectively (Jurica et al., 2001). Five subjects had unilateral GPi DBS, four subjects had bilateral GPi DBS, and one subject had bilateral STN DBS. All subjects were implanted with a Medtronic 3387 DBS lead and either an Activa SC, PC or RC IPG. All subjects complained of FOG before they received DBS surgery.

### Response to Stimulation

As part of the post-operative DBS programming protocol at UF, patients are assessed in the medication-OFF, DBS-OFF, and then DBS-ON state at the end of the 6-month optimization period. We analyzed this data to confirm that the subjects in this study were responsive to DBS to eliminate possible confounding factors. The mean (± SD) UPDRS part III motor scores in the medication-OFF/DBS-OFF vs. medication-OFF/DBS-ON state were 36.3 (± 11.9) and 25.2 (± 9.7), respectively (*p* = 0.001). The mean improvement from the DBS-OFF to the DBS-ON state was 52%.

### Safety and Tolerability

The BCDBS stimulation paradigm was safe and well tolerated by PD patients. Two subjects withdrew early from the study as they were unable to tolerate the OFF-medication time required for physical testing, however no adverse events from stimulation were experienced during the time they were participating in the study. There were technical difficulties with uploading the research firmware in one subject and we were unable to complete the testing protocol for that subject. However, the baseline programming settings were restored without difficulty. Expected stimulation induced side effects have been summarized in Table 1. The transient side effects all occurred immediately after turning the DBS ON from the DBS OFF state or immediately after modifying programming settings from one test condition to the next. One subject reported a persistent sensation of worsening balance with BCDBS, but this resolved with the return to their baseline settings. There were no permanent adverse effects or severe stimulation induced side effects that prevented participation in this trial. The biggest obstacle was limitation of physical activity in the medication-OFF state.

**Table 1:** Stimulation induced side effects during burst-cycling deep brain stimulation (BCDBS).

Side effects	4-Hz BCDBS (*n*)	10-Hz BCDBS (*n*)	15-Hz BCDBS (*n*)
Transient paresthesias	2	2	2
Transient concentration change	0	1	0
Persistent worsening balance^1^	1	1	1

*^1^Worsening of balance resolved upon reverting back to conventional deep brain stimulation (DBS)*.

### Clinical Outcomes

**Figure 3:**
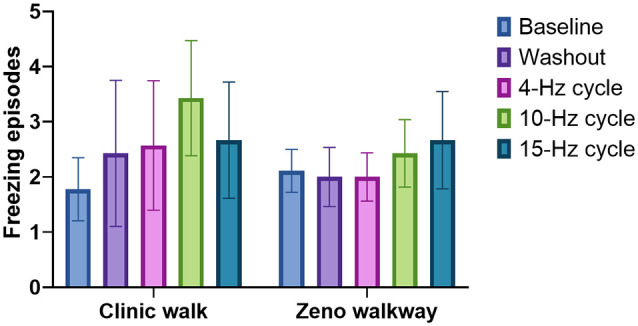
The effect of BCDBS on freezing of gait (FOG): the number of freezing episodes during two different walking tasks are shown for all five test conditions. There were no significant differences among all five groups.

A comparison of all five conditions for FOG is illustrated in Figure 3. Blinded video analysis of FOG episodes during the clinic walking trial and Zeno Walkway gait path revealed no significant difference among all testing conditions (*p* = 0.7480 and *p* = 0.9580 respectively). Single-leg-support percentage and double-leg-support percentage measured in the left and right leg using the Zeno Walkway gait analysis system identified no significant differences among all stimulation conditions (left single support *p* = 0.9820, left double support *p* = 0.9956; right single support *p* = 0.9115, and right double support *p* = 0.9942). Zeno Walkway assessment of temporal and spatial gait metrics showed no significant difference among all testing conditions for gait velocity and gait cadence (*p* = 0.9359 and *p* = 0.6854). Lastly, there was no difference detected among the testing conditions for the modified UPDRS part III motor scale and the timed up and go test (*p* = 0.9541 and *p* = 0.8984). The motor and gait outcomes are summarized in Supplementary Figures 1, 2.

## Discussion

FOG can be a debilitating symptom in PD and a challenge to treat *via* medication or neuromodulation (Huang et al., 2018). Given the associated increased risk of falls, FOG can have a significant impact on quality of life. In this study, we focused on evaluating the safety and feasibility of applying BCDBS for FOG in the setting of PD. We observed that BCDBS was safe and well tolerated. Stimulation induced side effects were transient, however one subject experienced a persistent sensation of worsening balance with BCDBS that resolved upon reverting back to conventional DBS. The most common feedback received during the testing protocol was difficulty engaging in physical activity in the medication-OFF state. There were no issues encountered with patient recruitment nor were there challenges with carrying out the testing protocol. The one instance of firmware technical difficulty was resolved by restoring the IPG back to manufacturer default settings. The observations from this study revealed that BCDBS could be safely applied in the clinic setting for future and potentially larger trials.

This study observed that BCDBS was non-inferior to conventional DBS for FOG, gait metrics, and motor symptoms in an acute setting. At the same time, BCDBS requires 88% less (at 4 Hz) to 54% (at 15 Hz) less battery consumption compared to conventional DBS at 130 Hz. As our collective understanding of neuromodulation expands along with evolving hardware and software capabilities, non-conventional DBS stimulation paradigms will likely continue to emerge. New paradigms could provide alternative solutions for DBS optimization in the context of sub-optimally placed leads, waning efficacy with disease progression, or difficult to treat symptoms (i.e., FOG).

It will be important to concurrently study the effects of new patterns of BCDBS at a brain-network level. Based on the stimulation-delivery paradigm, we hypothesized that BCDBS may confer electrophysiologic effects comparable to coordinated reset neuromodulation, variable frequency DBS or Temporally Optimized Patterned Stimulation (TOPS) DBS (Tass, 2003; Tass et al., 2012; Wilson and Moehlis, 2015; Jia et al., 2017). In this model, brief high-frequency pulses unlink the pathologic neuronal synchronization that is characteristic of the PD disease state (Tass et al., 2012). Other studies investigating the frequency-dependent effects of DBS have proposed that there is enhancement of inhibitory synaptic plasticity and frequency-dependent neuronal depression (Milosevic et al., 2018; Horn et al., 2020). Milosevic et al. (2018) observed a complex and dynamic temporal relationship with frequency-dependent stimulation. This suggests that there may be an optimal inhibitory plasticity state induced by neuromodulation and that advanced programming strategies may be able to achieve this.

Concurrent electrophysiology recordings enabled by new advances in hardware may also elucidate BCDBS effects on beta-band bursts and modulation (Adamchic et al., 2014). Future computational modeling studies could also investigate the effect of frequency for changes in whole brain connectomics. Popovych et al. (2017) demonstrated one such model utilizing a “pulsatile feedback stimulation” paradigm for a closed loop DBS system. Combining neuronal biophysical models with whole brain tractography may provide insight into specific pathways or targets that might most benefit from non-conventional DBS.

We acknowledge several limitations with this study. First, as this study was designed as a safety and tolerability study, a small patient cohort was evaluated in a within-subject crossover testing paradigm that was not counterbalanced. This introduces error into our clinical outcomes in the form of testing fatigue and prolonged medication-OFF time. Although there were no statistical differences among all testing conditions, trends that suggest 10-Hz and 15-Hz BCDBS had overall worse outcomes. This may be attributed to fatigue as 10-Hz and 15-Hz conditions were the last tests performed. Future studies utilizing a counterbalanced design in which the burst-cycling frequencies are tested in a randomized nonsequential order are needed to further explore this observation. Additionally, midway through the study our institution moved to a new facility. As a result, 3 out of 10 subjects were tested in a different clinic environment. This can affect the frequency of freezing episodes and gait metrics appreciated during the study. Furthermore, this study evaluated BCDBS in an acute setting. Observation of BCDBS in a chronic setting may be needed to elucidate any therapeutic effect. Lastly, this study was not designed to compare target-specific differential effects of BCDBS. A future prospective trial is needed to adequately evaluate if there are unique responses to BCDBS across the commonly used targets for FOG DBS.

In conclusion, BCDBS can be a safe and well tolerated novel stimulation paradigm. Future larger prospective studies will be needed to investigate the effectiveness of BCDBS and to understand the brain-network effects underpinning changes induced by this paradigm.

## Data Availability Statement

The raw data supporting the conclusions of this article will be made available by the authors, without undue reservation.

## Ethics Statement

The studies involving human participants were reviewed and approved by University of Florida Institutional Review Board. The patients/participants provided their written informed consent to participate in this study.

## Author Contributions

JW, RB, and LA: study conception and design. JW, WH, RB, JLo, PT, ZJ, AW, and LA: acquisition of data. JW, KM, JLe, AW, and TT: analysis and interpretation of data. JW and WH: drafting of manuscript. JW, WH, RB, JLo, JJ, RR, TT, MO, and LA: critical revision. All authors contributed to the article and approved the submitted version.

## Conflict of Interest

RR was employed by the company Medtronic. The remaining authors declare that the research was conducted in the absence of any commercial or financial relationships that could be construed as a potential conflict of interest.

## Supplementary Material

SUPPLEMENTARY FIGURE 1 Motor outcomes from burst-cycling deep brain stimulation (BCDBS): the mean duration and standard error of the **(A)** Timed Up and Go Test and **(B)** modified video Unified Parkinson’s Disease Rating Scale (UPDRS) part III are plotted for all five conditions in the medication-OFF state. There were no significant differences among all five conditions.

SUPPLEMENTARY FIGURE 2 Gait metrics from BCDBS: the mean and standard error of the **(A)** gait cadence, **(B)** gait velocity, and **(C)** single support/double support % for both legs are shown for all five conditions in the medication-OFF state. Gait metrics were recording using the Zeno walkway gait-analysis system. There were no significant differences among all five conditions. LSS, left single support; LDS, left double support; RSS, right single support; RDS, right double support.
