# Supplementary figures and images for: Safety and Tolerability of Burst-Cycling Deep Brain Stimulation for Freezing of Gait in Parkinson’s Disease
Source: Front Hum Neurosci. 2021 Apr 26;15:651168. doi: 10.3389/fnhum.2021.651168 (PMC8109241; doi:10.3389/fnhum.2021.651168)

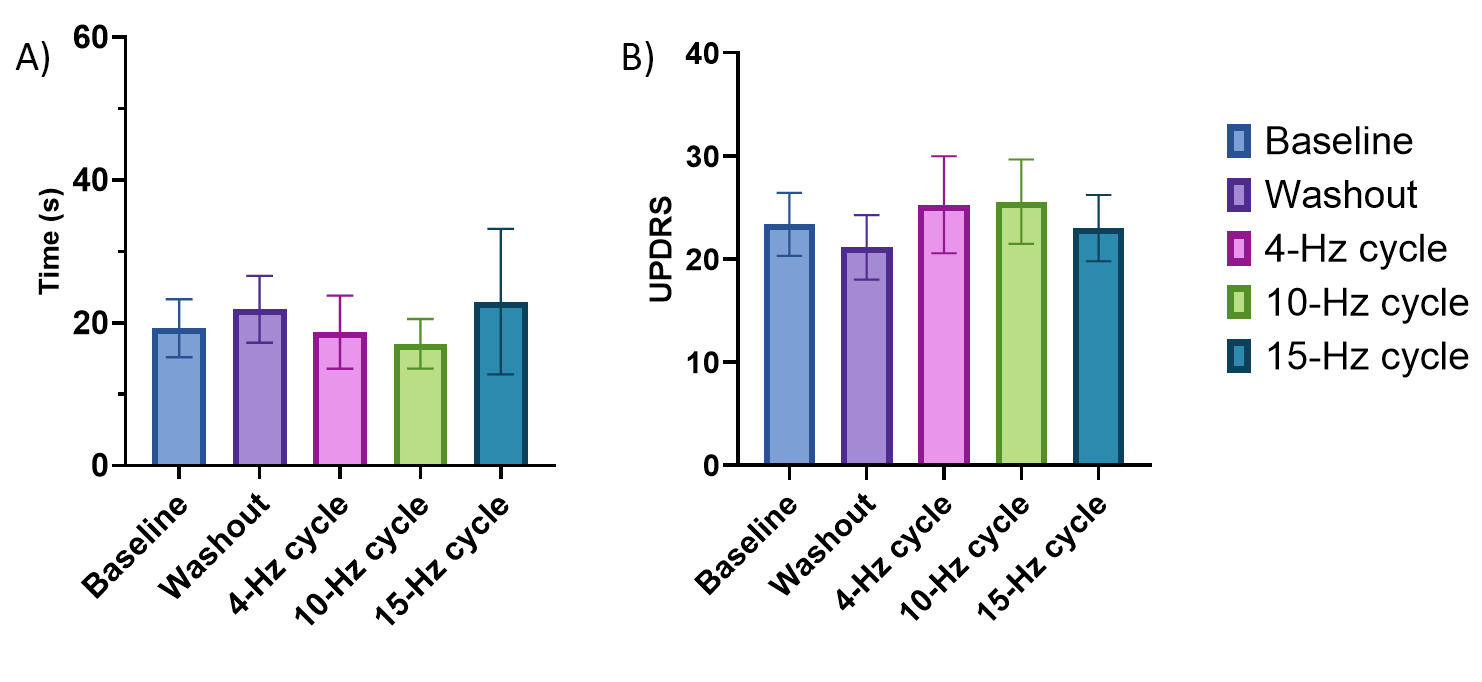

Supplement: SUPPLEMENTARY FIGURE 1 — Motor outcomes from burst-cycling deep brain stimulation (BCDBS): the mean duration and standard error of the (A) Timed Up and Go Test and (B) modified video Unified Parkinson’s Disease Rating Scale (UPDRS) part III are plotted for all five conditions in the medication-OFF state. There were no significant differences among all five conditions. [file Image_1.tif]

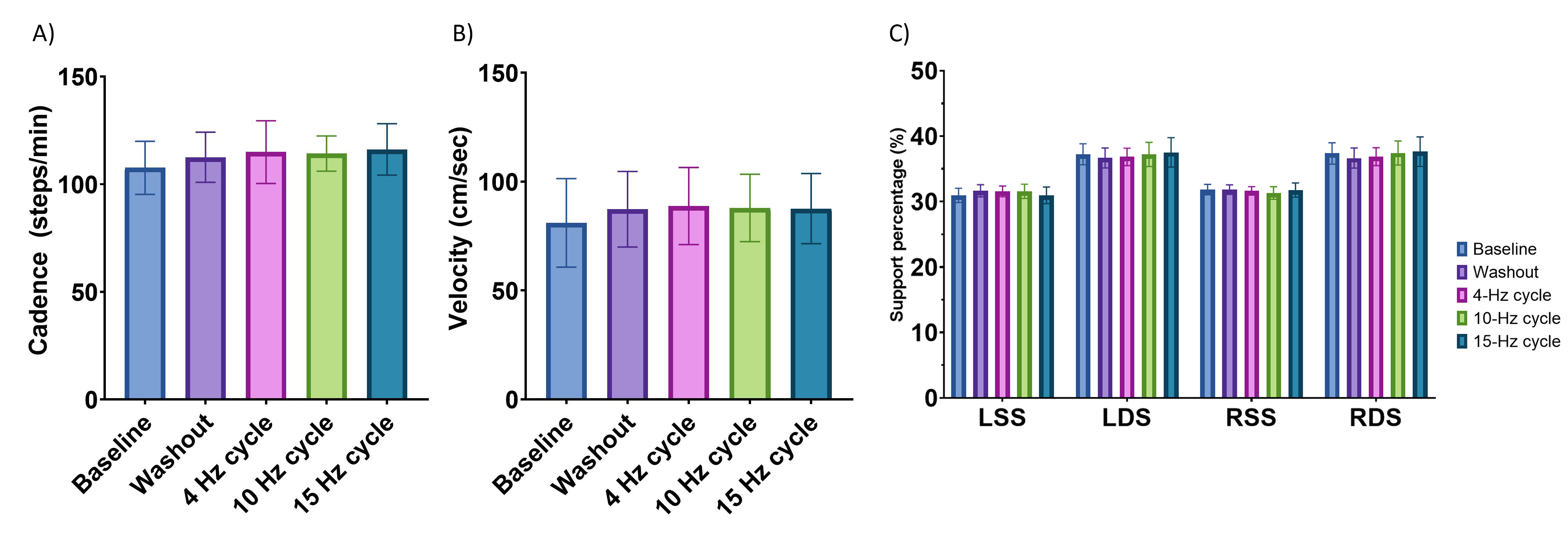

Supplement: SUPPLEMENTARY FIGURE 2 — Gait metrics from BCDBS: the mean and standard error of the (A) gait cadence, (B) gait velocity, and (C) single support/double support % for both legs are shown for all five conditions in the medication-OFF state. Gait metrics were recording using the Zeno walkway gait-analysis system. There were no significant differences among all five conditions. LSS, left single support; LDS, left double support; RSS, right single support; RDS, right double support. [file Image_2.tif]
